# Neonatal cardiac dysfunction and transcriptome changes caused by the absence of Celf1

**DOI:** 10.1038/srep35550

**Published:** 2016-10-19

**Authors:** Jimena Giudice, Zheng Xia, Wei Li, Thomas A. Cooper

**Affiliations:** 1 Department of Pathology and Immunology, Baylor College of Medicine, Houston, TX, 77030, USA; 2 Department of Cell Biology and Physiology, School of Medicine, University of North Carolina at Chapel Hill, NC, 27599, USA; 3 Department of Molecular and Cellular Biology, Baylor College of Medicine, Houston, Texas, 77030, USA; 4 Division of Biostatistics, Dan L Duncan Cancer Center, Baylor College of Medicine, Houston, TX, 77030, USA; 5 Department of Molecular Physiology and Biophysics, Baylor College of Medicine, Houston, TX, 77030, USA

## Abstract

The RNA binding protein Celf1 regulates alternative splicing in the nucleus and mRNA stability and translation in the cytoplasm. Celf1 is strongly down-regulated during mouse postnatal heart development. Its re-induction in adults induced severe heart failure and reversion to fetal splicing and gene expression patterns. However, the impact of Celf1 depletion on cardiac transcriptional and posttranscriptional dynamics in neonates has not been addressed. We found that homozygous Celf1 knock-out neonates exhibited cardiac dysfunction not observed in older homozygous animals, although homozygous mice are smaller than wild type littermates throughout development. RNA-sequencing of mRNA from homozygous neonatal hearts identified a network of cell cycle genes significantly up-regulated and down-regulation of ion transport and circadian genes. Cell cycle genes are enriched for Celf1 binding sites supporting a regulatory role in mRNA stability of these transcripts. We also identified a cardiac splicing network coordinated by Celf1 depletion. Target events contain multiple Celf1 binding sites and enrichment in GU-rich motifs. Identification of direct Celf1 targets will advance our knowledge in the mechanisms behind developmental networks regulated by Celf1 and diseases where Celf1 is mis-regulated.

CUGBP, Elav-like family member 1, Celf1, belongs to a family of RNA binding proteins containing six paralogs (Celf1-6). Celf1 and Celf2 are mainly expressed in heart, skeletal muscle, and brain^1^,^2^. Celf1 is highly conserved and is involved in multiple RNA processing functions. In the nucleus, Celf1 regulates alternative splicing, polyadenylation, and RNA editing. In the cytoplasm Celf1 controls mRNA stability, and translation^3^. CELF1 has been implicated in diverse human diseases including mis-regulation in several cancers^4^,^5^,^6^,^7^, up-regulation in Myotonic Dystrophy type 1 (DM1)^8^,^9^,^10^,^11^, and more recently it has been associated with Alzheimer disease^12^,^13^.

Celf1 protein expression patterns during development are conserved in the chicken and mouse^14^. In heart Celf1 protein expression levels are high during embryogenesis and the perinatal period, start to decrease at postnatal (PN) day 6–7, and at adult stages are dramatically reduced^14^,^15^. This developmental down-regulation of Celf1 protein correlates with coordinated alternative splicing transitions that occur between birth and adulthood^15^. We previously demonstrated that transgenic over-expression of human CELF1 specifically in cardiomyoctes in adult mice leads to severe cardiac failure. These animals exhibit extensive mis-regulation of alternative splicing and gene expression developmental networks^15^,^16^,^17^,^18^. Heart failure has been shown to induce a switch to fetal programs of alternative splicing^19^,^20^ and gene expression^21^. When Celf1 is re-induced in adults it is unclear which transcriptional and posttranscriptional changes are directly driven by Celf1 rather than an indirect effect of cardiomyopathy. We thus chose to use homozygous Celf1 knock out (*Celf1* −/−) mice to identify putative Celf1 pre-mRNA (splicing) and mRNA (stability) targets in mouse neonatal hearts.

Constitutive *Celf1* −/− mice have previously been shown to be viable when in a mixed strain background but with early mortality, growth retardation, and impaired fertility in surviving adults^22^. Here we aimed to identify the transcriptional and posttranscriptional networks regulated in heart by Celf1 using these *Celf1* −/− mice^22^. We were particularly interested in the perinatal period when Celf1 protein levels are relatively high prior to postnatal down-regulation. We first confirmed previous findings regarding the smaller size and reduced viability of homozygous mice^22^. We detected altered electrophysiological and contractile functions at early postnatal stages that could explain reduced viability. Animals did not show abnormal cardiac functions at five weeks of age. Deep RNA-sequencing (RNA-seq) of *Celf1* −/− heart samples identified extensive transcriptional changes at postnatal day 3 (PN3). We identified 45 alternative splicing events that are responsive to Celf1 depletion. Most of these events contain Celf1-CLIP tags and are enriched for GU rich motifs within the alternatively spliced regions and/or the flanking sequences suggesting that they are direct Celf1 splicing targets. Ion transport and circadian rhythm genes are significantly down-regulated in hearts from PN3 *Celf1* −/− animals in comparison with wild type PN3 hearts. Furthermore, we identified a network of cell cycle genes that are significantly up-regulated in PN3 *Celf1* −/− hearts. These genes are enriched for Celf1 binding sites based on CLIP-seq data supporting a regulatory role for Celf1 at neonatal stages in regulating the stability of mRNAs from cell cycle genes.

## Results

### Celf1 loss of function impacts viability

**Figure 1:**
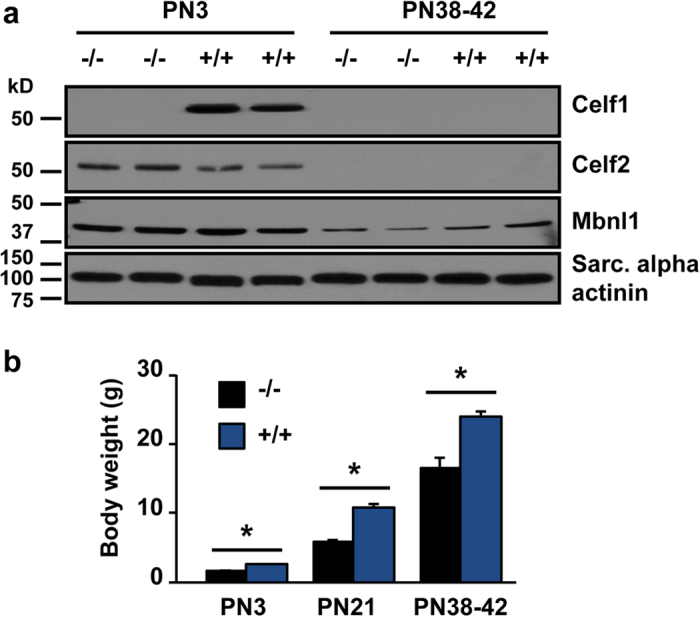
*Celf1* knock out impacts viability and animal size throughout postnatal development. (**a**) Celf1 protein levels were evaluated by Western blot assays at PN3 and PN38-42. Cropped blots are displayed. Full-length blots are shown in Supplementary Fig. S6. (**b**) Body weight was measured for PN3, PN21, and PN38-42. Results are shown as mean ± s.e.m. *p ≤ 0.05 Student t-test (2 tails), for PN3: *n* = 6 (3 females + 3 males) (*Celf1* −/−) and *n* = 8 (4 males + 4 females) (*Celf1* +/+), for PN21: *n* =  4 (2 females + 2 males) (*Celf1* −/−) and *n* = 12 (6 males + 6 females) (*Celf1* +/+), for PN38-42: *n* =  3 (2 females + 1 male) (*Celf1* −/−) and *n* = 3 males (*Celf1* +/+) Sarc: sarcomeric.

Celf1 protein is down-regulated more than ten times during postnatal heart development^15^. Therefore, we first evaluated Celf1 protein expression levels in hearts at neonatal (PN3) and later (PN38-42) stages from *Celf1* −/− and *Celf1*+/+ animals by Western blot assays. Celf1 protein was completely absent in hearts from *Celf1* −/− neonates (Fig. 1a). Celf1 mRNA levels at PN3 were decreased 20-fold in *Celf1* −/− hearts based on RNA-seq data. Celf1 protein expression in adult (PN38-42) *Celf1* +/+ animals decreased approximately 40-fold and was not detected in *Celf1* −/− mice (Supplementary Fig S1). Only a slight up-regulation of the paralog Celf2 was observed and there was no change in level of Mbnl1, an RNA binding protein that co-regulates a subset of Celf1 targets (Fig. 1a).

As described previously^22^, *Celf1* −/− animals were smaller than their wild type littermates throughout postnatal development (Fig. 1b) and the number of three-four week old *Celf1* −/− animals from *Celf1* −/+ matings was significantly lower than expected. At PN3, we observed that out of 75 mice (from ten Celf1 −/+ matings), 42 were heterozygous, 21 were wild type, and only 12 were homozygous. On the other hand, out of 107 PN21-28 mice from 15 Celf1 −/+ matings, 65 were heterozygous, 32 were wild type, and only 10 were homozygous (Supplementary Table S1). For litters evaluated at PN3, *χ*^2^ did not reach significance, (Supplementary Table S1) although the data showed a trend toward a lower than expected number of Celf1 −/− animals. However, *χ*^2^ was significant (*p* < 0.001) at PN21-28 demonstrating that fewer homozygous animals than expected survived (Supplementary Table S1). Taken together and consistent with a previous report^22^, we conclude that Celf1 loss of function leads to retarded growth and reduced viability.

### Cardiac function is significantly affected in *Celf1* −/− neonates

**Figure 2:**
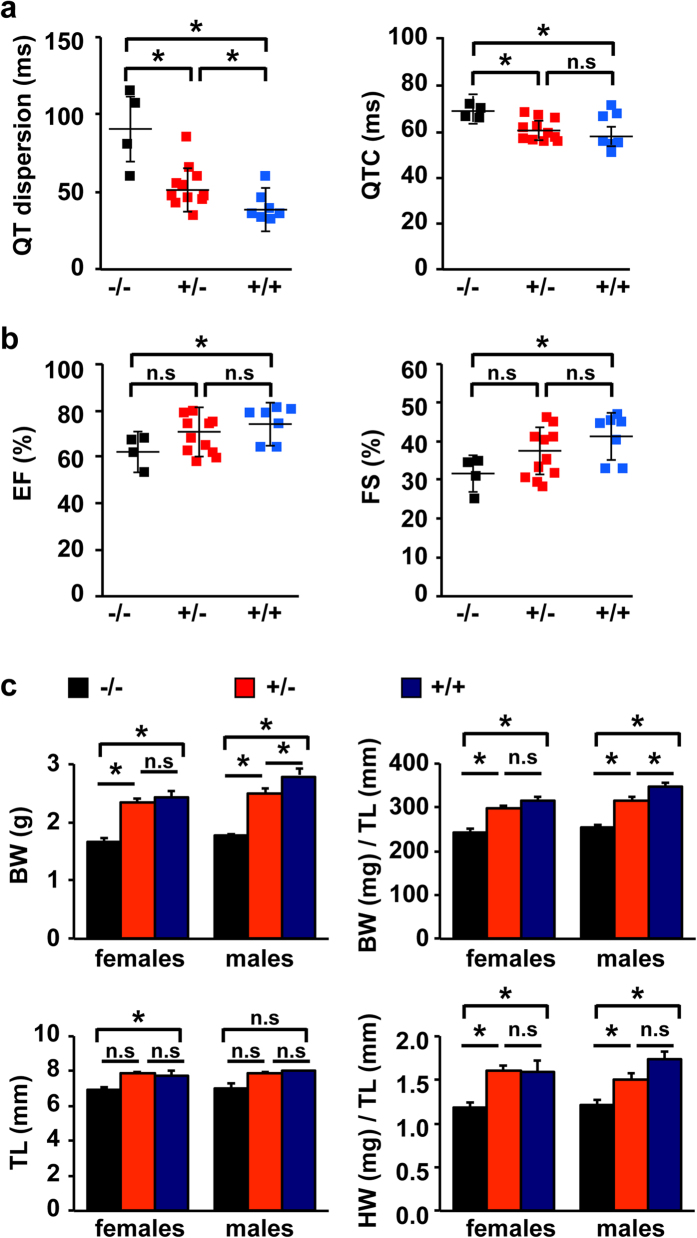
Cardiac functions and heart and body weights are reduced in *Celf1* −/− neonates. (**a**) QT dispersion and QTc were measured by ECG assays in neonates at PN3-5. See Supplementary Table S4. (**b**) Ejection fraction (EF) and fraction shortening (FS) were evaluated by echocardiograms in neonates at PN3-5. See Supplementary Table S5. Results are shown as mean ± s.e.m. *p ≤ 0.05 Student t-test (2 tails), *n* = 4 (*Celf1* −/−), *n* = 11 (*Celf1* +/−), *n* = 7 (*Celf1* +/+). (**c**) Body and heart weights (BW and HW, respectively) and tibia length (TL) were measured in neonates at PN3. Results are shown as mean ± s.e.m. *p ≤ 0.05 Student t-test (2 tails). Females: *n* = 4 (*Celf1* −/−), *n* = 13 (*Celf1* +/−), *n* = 4 (*Celf1* +/+). Males *n* = 3 (*Celf1* −/−), *n* = 12 (*Celf1* +/−), *n* = 6 (*Celf1* +/+). n.s: not significant.

We next examined cardiac functions at two developmental stages, early after birth (PN3-5) and in young adults (PN35-36). Echocardiogram and electrocardiogram (ECG) studies showed no significant differences between homozygous and wild type animals at PN35-36 (Supplementary Tables S2 and S3). However, at neonatal stages QTc (corrected QT interval) and QT dispersion (maximum QT interval minus minimum QT interval) were increased in *Celf1* −/− animals reflecting abnormalities in repolarization^23^ and disparity of ventricular recovery times^24^ (Fig. 2a, Supplementary Table S4). The volumetric fraction of blood pumped out of the left and right ventricles per heart-beat is known as the ejection fraction (EF). EF was reduced in *Celf1*−/− animals (62% ± 3%, *n* = 4) in comparison with heterozygous (69% ± 2%, *n* = 11, *p* = 0.18 homozygous versus heterozygous), and wild type mice (75% ± 3%, *n* = 7, *p* = 0.03 homozygous versus wild type) (Fig. 2b, left, Supplementary Table 5). Similarly, the shortening level of left the ventricular diameter between end-diastole and end-systole (fractional shortening, FS) was significantly reduced in homozygous animals (31% ± 2%, *n* = 4) in comparison with heterozygous (36% ± 2%, *n* = 11, *p* = 0.12 homozygous versus heterozygous), and wild type mice (41% ± 2%, *n* = 7, *p* = 0.02 homozygous versus wild type) (Fig. 2b, right, Supplementary Table S5). The other echocardiographic parameters showed no differences between genotypes and M-mode images (Supplementary Fig. S2) from each genotype are consistent with the quantitative data presented in Fig. 2b and Supplementary Table S5 in that there is little difference between the genotypes in the gross anatomic changes during contraction.

We studied more in detail the neonatal animals measuring their body weight, heart weight and tibia length. We normalized heart weights and body weights to tibia length since we observed differences in body weight. Overall, we observed that both PN3-5 homozygous females and males exhibited significant smaller body and heart weights than wild type animals (Fig. 2c).

In conclusion we found that neonate cardiac functions and morphology are impacted by the absence of Celf1. Hearts of PN3-5 *Celf1* −/− pups are smaller and show reduced function compared to those from wild type and heterozygous littermates, possibly contributing to reduced viability.

### Celf1 regulates alternative splicing in neonatal hearts

**Figure 3:**
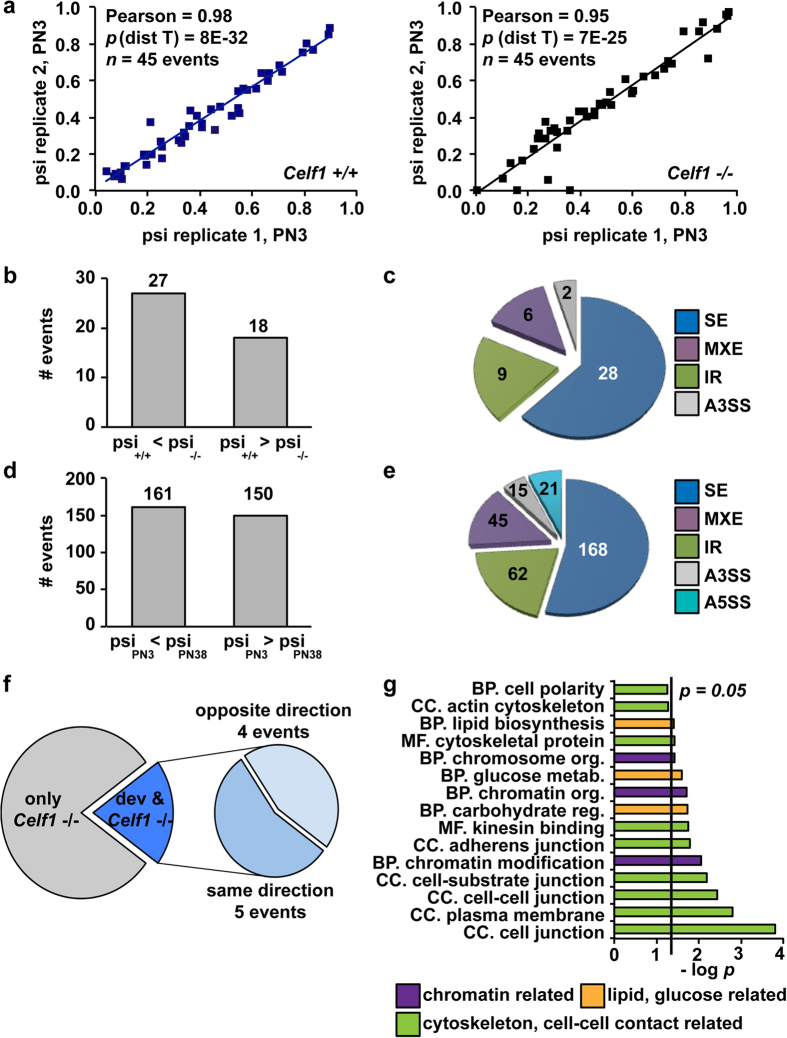
Celf1 loss of function results in alternative splicing changes in neonatal hearts. (**a**) Analysis of duplicate RNA-seq data sets (PN3) showed high levels of reproducibility evaluated by Pearson coefficients. (**b,c**) Number (**b**) and type (**c**) of alternative splicing events responsive to Celf1 absence at PN3. (**d,e**). Number (**d**) and type (**e**) of alternative splicing events regulated during development in wild type animals. (**f**) Alternative splicing events responsive to Celf1 absence at PN3 and also developmentally regulated between PN3 and PN38. (**g**) Gene ontology analysis of splicing events responsive to Celf1 absence in neonates (PN3). BP: biological processes. CC: cellular components. Dev: development. MF: molecular functions. Org: organization. Reg: regulation.

To identify the transcriptional and posttranscriptional networks regulated by Celf1 in neonatal hearts, we performed RNA-seq using polyadenylated mRNA isolated from ventricles of *Celf1* −/− and *Celf1* +/+ animals at PN3 and from *Celf1* +/+ animals at PN38 at which point Celf1 levels have decreased (Supplementary Fig. S1). The high quality of the RNA-seq data was reflected by efficient mapping to the genome for all samples (85–86%) and sufficient depth for alternative splicing analysis (>179,000,000 paired end reads) (Supplementary Table S6). Alternative splicing data (percent spliced in, PSI) from two replicates of homozygous or two wild type samples showed high levels of correlation (Pearson = 0.98 for *Celf1* +/+ animals, Pearson = 0.95 for *Celf1* −/− animals) (Fig. 3a).

We identified 45 alternative splicing events affected in *Celf1* −/− neonatal heart (|Δpsi| ≥ 15%) (Supplementary Table S7). Out of the 45 events, 27 (60%) exhibited more inclusion in *Celf1* −/− compared to wild type animals (psi_*Celf1*+/+_ < psi_*Celf1/−*_) and 18 (40%) transitions showed the opposite change (psi_*Celf1*__+/+_ > psi_*Celf1/−*_) (Fig. 3b). The affected alternative splicing events were mainly cassette exons (28 events, 62%) and a lower proportion of them were intron retention (IR) (nine events, 20%), mutually exclusive exons (MXE) (six events, 13%), and alternative 3′ splice site selection (A3SS) (two events, 4%) (Fig. 3c). On the other hand, alternative splicing events regulated during postnatal development (PN3 to PN38) were more equally distributed in terms of skipping/inclusion: 161 events (52%) showed more inclusion at PN38 than in neonates, and 150 events (48%) showed more skipping at PN38 (Fig. 3d). Developmentally regulated splicing events were also mainly cassette exons (168 events) and 62 IR events, 45 MXE, 15 A3SS, and 21 alternative 5′ splice sites (A5SS) (Fig. 3e).

From the 45 alternative splicing events responsive to Celf1 depletion at PN3 only nine events were also regulated during development between PN3 and PN38 when Celf1 levels decrease (five of them in the same direction as development and four in the opposite direction) (Fig. 3f). Gene ontology analysis of the genes with alternative splicing changes in PN3 *Celf1*−/− animals revealed that the most significantly enriched categories were related to chromatin organization (purple), cytoskeleton functions and cell-cell contact (green), and lipid and glucose metabolism (yellow) (Fig. 3g).

### Alternative splicing events sensitive to Celf1 depletion contain Celf1 CLIP tags and are enriched in GU rich motifs

**Figure 4:**
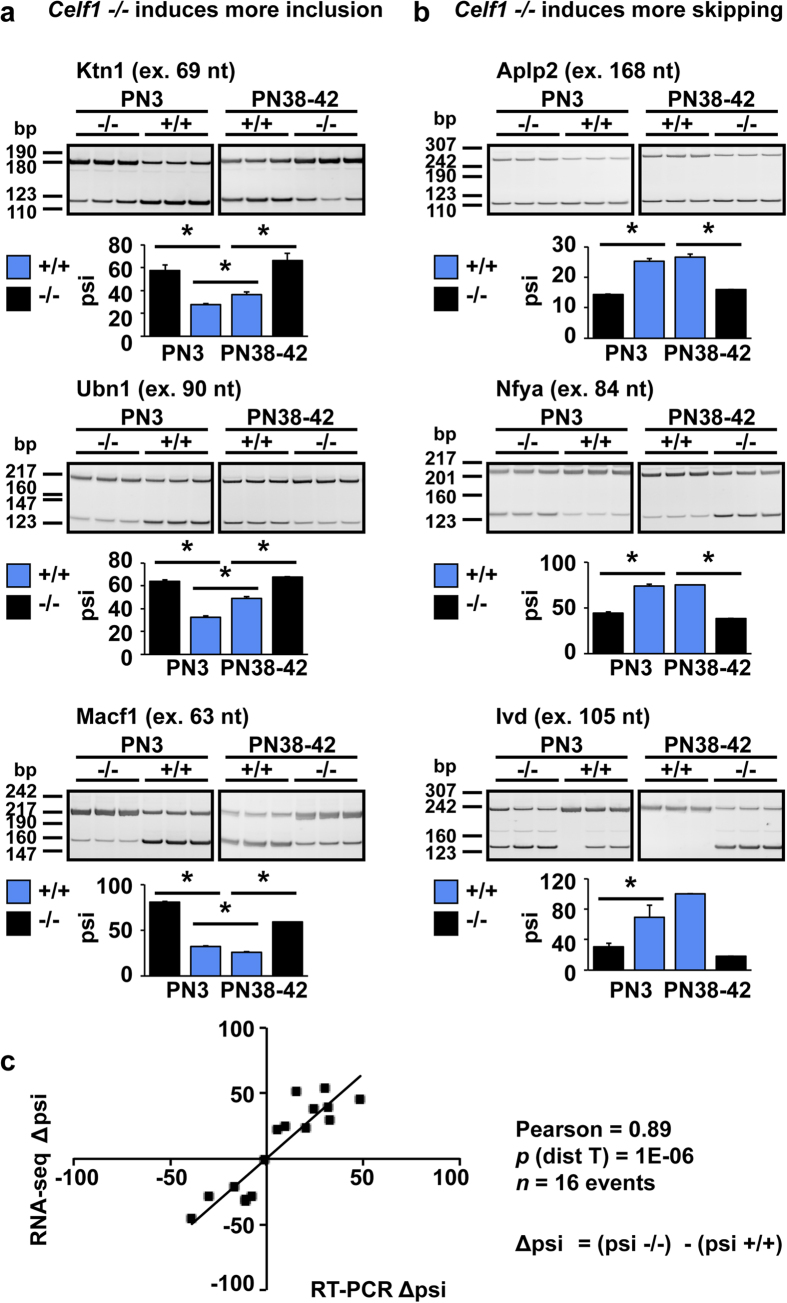
Validation of neonatal alternative splicing changes in the absence of Celf1. (**a,b**) Celf1 responsive splicing events identified by RNA-seq were validated by RT-PCR assays in RNA from *Celf1*−/− and *Celf1*+/+ hearts at PN3 and PN38-42. Left panel (**a**) shows three examples of events where Celf1 deletion induced more inclusion and right panel (**b**) three examples where Celf1 deletion promoted skipping. Other examples are shown in Supplementary Fig. S3. Results are shown as mean ± s.e.m. *p ≤ 0.05 Student t-test (2 tails), *n* = 3. (**c**) Correlation analysis between Δpsi values (psi_*Celf1−/−*_ - psi_*Celf1*+/+_) obtained by RNA-seq and RT-PCR for 16 splicing events at PN3 (see Supplementary Table S8). Cropped gels are displayed. Full-length gels are shown in Supplementary Fig. S7.

We next validated the alternative splicing data by reverse transcription (RT) PCR experiments. We designed primers annealing in the constitutive flanking regions of 16 alternative regions regulated by Celf1 depletion at PN3 (Supplementary Table S8). We performed RT-PCR using heart RNA from *Celf1* +/+ and *Celf1*−/− animals at PN3 and PN38–42 (Fig. 4a,b, and Supplementary Fig. S3). The correlation between Δpsi (psi_*Celf1*−/−_ - psi_*Celf1*+/+_) at PN3 obtained by RT-PCR and by RNA-seq was high (Pearson coefficient = 0.89) confirming the validity of quantitative splicing analysis from the RNA-seq data (Fig. 4c and Supplementary Table S8).

Celf1 binds GU rich motifs in regions in close proximity to the regulated splicing event^25^. We thus analyzed the motifs enriched in 22 alternative regions and 500 bp up- and downstream using MEME software^26^. A GU rich motif was most significantly enriched being present in 16 out of the 22 splicing events analyzed (Supplementary Fig. S4a,b). Interestingly a GA rich motif was also enriched (Supplementary Fig. S4a). Motif enrichment analysis was used as a first approach to investigate the potential splicing targets of Celf1. However, to better evaluate direct Celf1 effects on alternative exons we looked for already available experimental evidence of Celf1 binding to pre-mRNAs. High-throughput sequencing of RNA isolated by crosslinking immunoprecipitation (HITS-CLIP)^27^ allows global identification of targets for specific RNA binding proteins in cells. We used a Celf1 HITS-CLIP data set from the mouse C2C12 cell line^28^ to analyze Celf1-CLIP tags within or in close proximity to the alternative splicing events sensitive to the absence of Celf1 in neonatal hearts. Out of the 45 events we found that 31 (69%) contained at least one Celf1-CLIP tag within the alternative region +/− 500 bp up- or downstream (Supplementary Fig. S4c, left). The presence of Celf1-CLIP tags was significantly higher (*p* = 0.001) within +/− 500 bp around alternative spliced regions affected by *Celf1* −/− than around 50 alternative splicing events responsive only to *Mbnl1* −/− (Supplementary Fig. S4c, right). Four events (9%) contained Celf1-CLIP tags in the flaking 500–800 bp, and the remaining 10 events (22%) did not contain any Celf1-CLIP tag within the alternative region or the flanking sequences (+/−800 bp) (Supplementary Fig. S4c).

In summary, we identified alternative splicing events that are likely to be direct Celf1 targets in neonatal mouse heart. This conclusion is mainly based on the fact that they *i)* respond to Celf1 depletion in neonatal hearts, and *ii)* contain Celf1-CLIP tags within the alternative exons and/or the flanking regions.

### Cell cycle gene mRNAs are up-regulated in PN3 *Celf1*−/− hearts and are enriched for Celf1 CLIP tags

**Figure 5:**
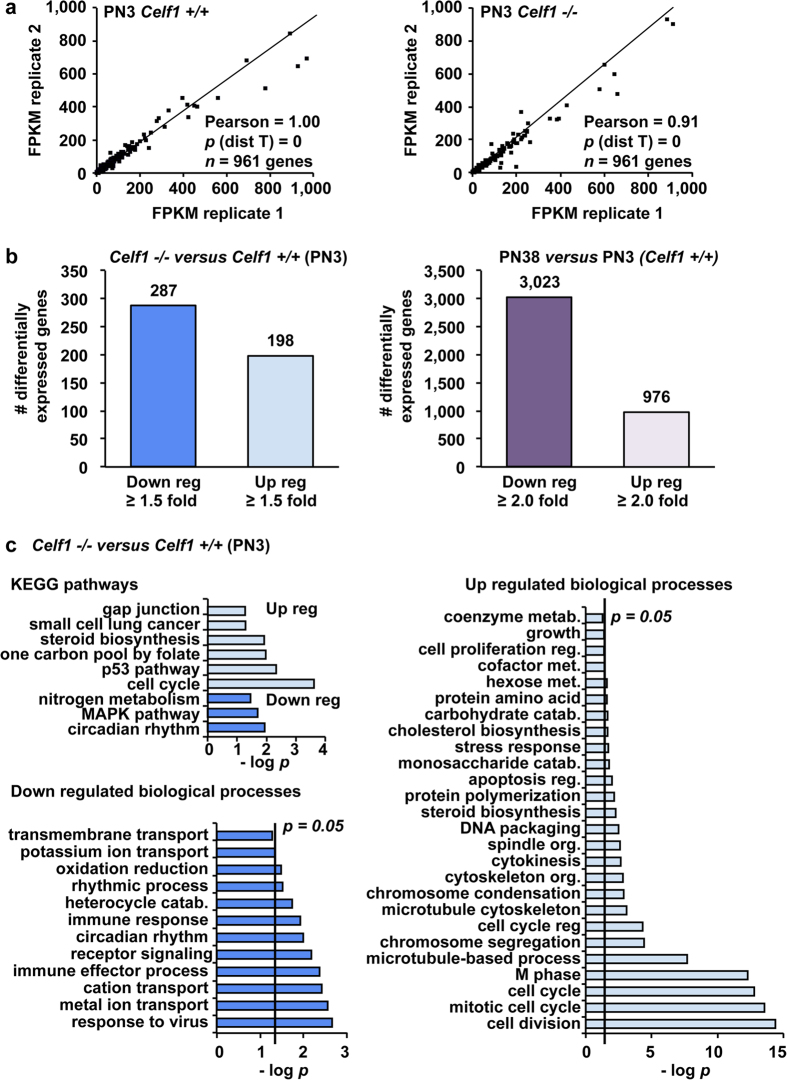
Celf1 loss of function correlates with altered expression of mRNAs from cell cycle, ion transport and circadian genes. (**a**) Analysis of duplicate RNA-seq data sets (PN3) showed high levels of reproducibility evaluated by Pearson coefficients. (**b**) Genes differentially expressed ≥ 1.5 fold (FDR ≤ 0.05) (PN3 *Celf1* −/− *vs Celf1* +/+) or ≥ 2.0 fold (FDR ≤ 0.05) (PN38 versus PN3 wild type animals). (**c**) Gene ontology analysis of differentially regulated genes in *Celf1* −/− animals in comparison with *Celf1* +/+ was performed using DAVID software (biological processes and KEGG pathways) (See Supplementary Tables S10 and S11). Catab: catabolism. Down reg: down regulated. Metab: metabolism. Org: organization. Reg: regulation. Up reg: up regulated.

RNA-seq revealed a large number of genes differentially expressed in the absence of Celf1 in neonatal hearts (Supplementary Table S9). Correlation analysis of the gene expression levels expressed as FPKM (fragments per kilobase of transcript per million mapped reads) for *Celf1* −/− and *Celf1* +/+ biological replicates at PN3 showed high Pearson coefficients (1.00 and 0.91, respectively) demonstrating reproducibility (Fig. 5a). We next analyzed the number of transcripts differentially expressed (≥1.5 fold, with FDR ≤ 0.05) in *Celf1* −/− compared to *Celf1* +/+ hearts at PN3 and those differentially expressed during heart development between PN3 and PN38 in *Celf1* +/+ mice (≥2.0 fold, with FDR ≤ 0.05; threshold differences are due to the extensive number of genes developmentally regulated). There were 483 genes differentially expressed in *Celf1* −/− compared to *Celf1* +/+ in PN3 hearts (287 down-regulated and 198 up-regulated) (Fig. 5b, left). During *Celf1* +/+ mouse postnatal heart development 3,023 genes were down-regulated and 976 genes were up-regulated (Fig. 5b, right).

**Figure 6:**
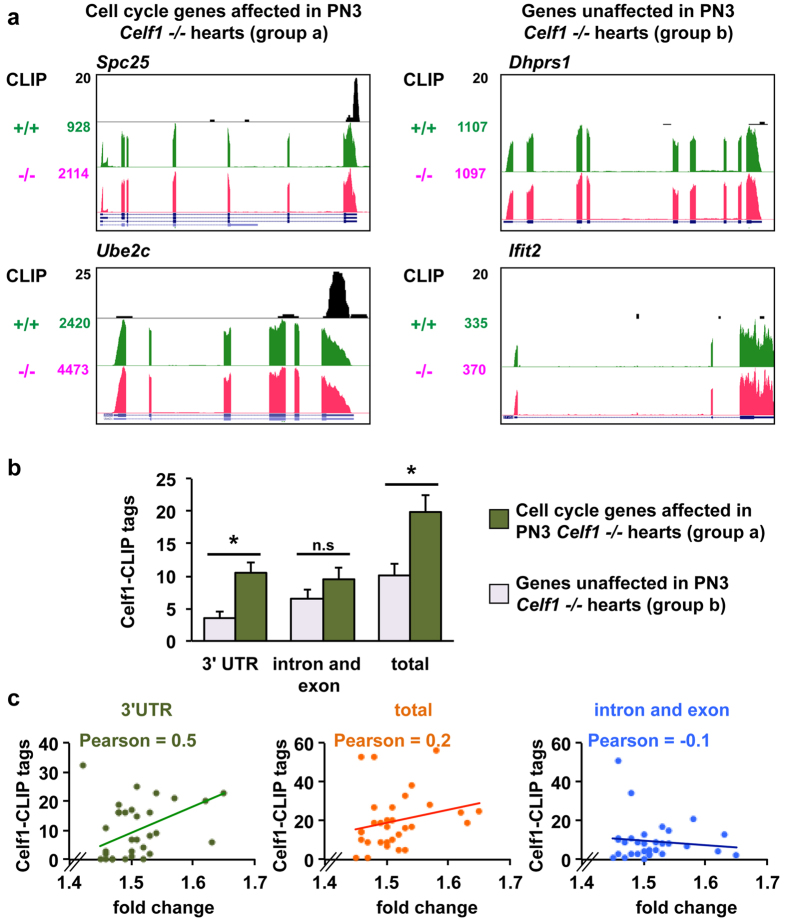
Cell cycle gene mRNAs that are sensitive to Celf1 depletion are enriched for Celf1 CLIP tags. (**a**) Celf1-CLIP tags and RNA-seq data were visualized using the UCSC genome browser. Two examples of cell cycle genes regulated by Celf1 depletion (left) and two for Celf1-nonresponsive genes (right) are shown. (**b**) Celf1-CLIP tags located within the 3′UTRs and those located within intronic and exonic regions were computed for all 31 genes included in the “cell cycle” gene category (group a) and randomly selected 31 genes non responsive to Celf1 absence (group b) (Supplementary Table S12 left side shows group a and right side shows group b). Total Celf1-CLIP refers to the sum of those in the 3′UTRs and those in the intron and exon regions. Results are shown as mean ± s.e.m. *p ≤ 0.05 Student t-test (2 tails). n.s: not significant. **(c**) Correlation graphs between the Celf1-CLIP tags and up-regulation levels (fold change) (3′UTRs, the exonic and intronic regions, and total).

Global gene ontology analysis of the genes differentially expressed in *Celf1* −/− hearts at PN3 revealed a significant enrichment in functions related to cell cycle and proliferation (up-regulated) and ion transport, immune response, and circadian rhythm (down-regulated) (Fig. 5c and Supplementary Tables S10 and S11). Celf1 has been shown to bind 3′ UTRs of transcripts and regulate mRNA stability^28^,^29^,^30^. To test the hypothesis that the absence of Celf1 in PN3 hearts affected mRNA levels of cell cycle genes we evaluated the presence of Celf1-CLIP tags within all of the 31 genes listed in the cell cycle category that was one of the most significant and the category with the highest number of genes (cell cycle, *p* = 2E-31) (Supplementary Table S10). We analyzed two groups of genes side by side: *i)* the 31 cell cycle genes (group a), and *ii)* a set of randomly selected 31 genes that did not show mRNA expression changes in *Celf1*−/− mice (group b). Visual inspection of all 62 genes using the UCSC genome browser revealed that cell cycle genes (group a) contained more Celf1-CLIP tags per gene in their 3′ UTRs in comparison with genes that were not affected by Celf1 loss (group b) (Fig. 6 show two examples of each group).

This observation led us to systematically compute the Celf1-CLIP tags present within the 3′ UTRs and those located within intronic and exonic regions for each of the cell cycle genes of group a (left side of Supplementary Table S12). We performed similar analysis in the 31 genes randomly selected that were unaffected by Celf1 deletion (group b) (right side of Supplementary Table S12). The analysis revealed that Celf1-CLIP tags are significantly more prevalent in 3′UTRs of the regulated cell cycle genes (group a) compared to the set of control genes (group b) that are not affected by Celf1 loss of function (Fig. 6b). There were no significant differences in the presence of Celf1-CLIP tags within intronic or exonic regions of these two sets of genes (group a *versus* group b) however the total number of CLIP tags (3′UTR + introns and exons) was significantly different reflecting the differences in the 3′UTRs (Fig. 6b). In addition the correlation between Celf1-CLIP tags and the level of up-regulation (fold change) was higher for the 3′UTRs (Pearson = 0.5) than for the intronic or exonic regions (Pearson = −0.1) and total (Pearson = 0.2) (Fig. 6c) consistent with a correlation between Celf1 binding and mRNA levels.

Although our experiments and data cannot rule out other mechanistic scenarios, one possible explanation is that Celf1 may regulate the mRNA stability of a subset of cell cycle genes by binding to the 3′UTRs. Assuming the hypothesis that Celf1 binding promotes mRNA decay^31^, mRNAs would be stabilized in the absence of Celf1 consistent with the observed mRNA up-regulation in PN3 *Celf1*−/− hearts. Further molecular experiments will be necessary to confirm this hypothesis and/or identify more complex mechanistic explanations for our global and high-throughput findings.

## Discussion

We have characterized the *Celf1* −/− mice complementing previous work from Luc Paillard and colleagues^22^,^32^. While past studies focused on the physiological impact of Celf1 depletion in growth, spermatogenesis, and skeletal muscle, we provide characterization of the cardiac features in *Celf1*−/− neonatal heart when Celf1 protein is normally high. Celf1 is a RNA binding protein that orchestrates multiple transcriptional and posttranscriptional programs important in normal development and disease. The mis-regulated expression of CELF1 seen in several diseases highlights the potential physiological importance of these coordinated networks. While the transcriptional and posttranscriptional effects of CELF1 over-expression have been studied *in vivo*^15^,^16^,^17^, the effects of Celf1 loss of function have not. This is particularly important in cardiac biology because Celf1 is down-regulated during postnatal development and its up-regulation in adult cardiomyocytes leads to heart failure^15^,^16^,^18^.

The alternative splicing events that we found to be responsive to the absence of Celf1 in PN3 heart were also enriched for Celf1-CLIP tags within the variable region or in the flanking sequences together with GU rich preferred binding motifs for Celf1. These data suggest that the identified splicing events are likely to be direct Celf1 targets. Many of these splicing events are regulated by Celf1 depletion in C2C12 cells (unpublished data), reducing concern of secondary effects *in vivo*.

In terms of gene expression, it is interesting to note that it was previously shown that a dominant negative CELF protein expressed in heart produced a mild phenotype in young animals that resolved with aging^33^. Similarly to that study, our results also described the spontaneous recovery of a heart phenotype. Overlapping analyses indicate that only three of the genes affected by the mild or severe CELF1 dominant negative were also affected in the *Celf1*−/− PN3 hearts using the 2.0 fold change in gene expression. For all three, the gene expression was affected in the same direction for the dominant negative and *Celf1*−/− hearts consistent with a loss of function. However the relatively small number of genes precludes making firm conclusions.

We identified a set of cell cycle genes that are up-regulated in the absence of Celf1 in PN3 hearts. We also provided evidence that the 3′UTRs of the cell cycle gene mRNAs are enriched for Celf1 binding sites. This binding is expected to destabilize the mRNA and therefore the absence of Celf1 at early neonatal stages is expected to stabilize cell cycle transcripts consistent with the up-regulation we observed. In heart and other tissues such as liver and brain, cell cycle and cell proliferation genes are strongly down-regulated after birth to allow cell differentiation and tissue maturation for adult functions^16^,^34^,^35^. The fact that cell cycle genes are up-regulated when Celf1 is absent at PN3 together with the Celf1-CLIP tag presence in their 3′UTRs is compatible with the idea that Celf1 is exerting a destabilizing role on those transcripts. However, we cannot rule out other molecular mechanisms involved in the up-regulation of cell cycle genes observed in neonatal hearts when Celf1 is absent. Among these other mechanisms we should mention regulators acting in concert with Celf1 and transcriptional modifiers (transcription factors, epigenetics modifiers, etc.) regulated by Celf1 depletion that are the direct drivers of the changes in cell cycle genes. In addition, during normal postnatal development Celf1 is down-regulated^15^ as are the cell cycle genes^16^ leading to an apparent discrepancy However, postnatal heart development is highly dynamic with regard to gene expression changes^16^ (Fig. 5). This apparent discrepancy can be likely due to a scenario in which Celf1 contributes to maintaining a level of mRNAs by acting in combination with additional regulatory factors that become dominant repressors of mRNA, chromatin or transcription in the postnatal to adult transition. Although our work open important new molecular questions to address experimentally, this work provides a new high-throughput view of the transcriptional dynamics in the absence of Celf1 in neonatal hearts.

## Methods

### Animals

All animals were handled following the NIH Guidelines for Use and Care of Laboratory Animals that were approved by the Institutional Animal Care and Use Committee (IACUC) at Baylor College of Medicine. Dr. Luc Paillard from the Centre National de la Recherche Scientifique (CNRS, France) kindly provided us the *Celf1* −/+ heterozygous mice (mixed 129Sv/BL6 strain)^22^. Animals were anesthetized before cervical dislocation (older than PN21) or decapitation (neonatal) and the hearts were removed. Blood and atria were carefully removed and the ventricles were flash frozen in liquid nitrogen and kept at −80 °C until use. Littermates were used for comparisons between genotypes for physiological and molecular analyses to standardize differences between animals and reduce variability as much as possible due to the mixed strain background. While we did not perform a formal survival analysis, an analysis of animals not selected for euthanasia provides a sense of reduced *Celf1*−/− survival detected by PN3 suggesting either reduced fetal or neonatal survival and a fraction of animals survive beyond two months of age (Supplementary Fig. S5).

### Western blot assays

Ventricles were lysed in HEPES-sucrose buffer (10 mM HEPES pH 7.4, 0.32 M sucrose, 1 mM EDTA, and proteases inhibitors) using Bullet blender (Next Advance). SDS was added after tissue disruption until a final concentration of 1%. Samples were sonicated for 3 min at 75 V (30 s on, 30 s off) and centrifuged for 10 min at 14,000 r.p.m at 4 °C. Supernatants were transferred into new tubes and the protein concentration was estimated utilizing the Pierce BCA protein assay kit (Thermo Scientific #23225). Samples were diluted in loading buffer (100 mM Tris-HCl pH 6.8, 4% SDS, 0.2% Bromophenol blue, 20% glycerol, 200 mM β-mercaptoethanol) and boiled for 5 min. A total amount of 40 μg protein was separated by 10% SDS-PAGE and transferred into nitrocellulose membranes for 2 h at 120 V. Membranes were blocked with 5% non-fat dried milk in 0.1% Tween-TBS buffer (T-TBS) for 1 h, washed and incubated overnight at 4 °C with primary antibodies diluted in 5% milk/T-TBS: mouse monoclonal anti-CUG-BP1, clone 3B1 (Milipore, #05-621) (1:1,000), mouse monoclonal anti-MBNL1 (Life Span #LS-B4372), a home made mouse monoclonal anti-Celf2 antibody (Etr3 clone 1H2)^36^ (1:1,000), rabbit polyclonal anti-sarcomeric alpha actinin (Abcam, #ab72592) (1:2,000). The next day, membranes were incubated with the secondary antibodies (1:5,000) diluted in 5% milk/T-TBS for 1 h at room temperature. The secondary antibodies were a peroxidase-conjugated goat anti-mouse IgG light chain specific (Jackson Immunoresearch, #115-035-174) and goat anti-rabbit IgG horseradish peroxidase-conjugated (Invitrogen, # 621234). Membranes were developed using the Super Signal West Pico Chemiluminiscent Substrate kit (Thermo Scientific #34080).

### RNA isolation

RNA was extracted using the RNeasy fibrous tissue mini-kit (Qiagen #74704) following manufacturer protocols.

### Alternative splicing validation by RT-PCR

RT reactions were performed using the High Capacity cDNA RT Kit (Applied Biosystem #4368814) and PCRs using GoTaq DNA Polymerase (Promega #M7123). In both cases, manufacturer protocols were followed. PCR program contained the following steps: *(i)* 95 °C for 1 min 45 s, *(ii)* 28 cycles of 95 °C for 45 s, 57 °C for 45 s and 72 °C for 1 min, *(iii)* 72 °C for 10 min, and *(iv)* 25 °C for 5 min. Primer sequences (Sigma) for the alternative splicing events evaluated are described in Table S8. PCR products were analyzed by 6% PAGE. We quantified the percentage spliced in (psi)^37^ of the alternative regions by densitometry using ImageJ plugin for gel analysis and following equation 1 (Eqn 1).


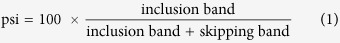


### Genotyping

DNA was extracted from tail clips using Direct PCR lysis reagent (Viagen #102-T) following manufacturer protocols. PCR reactions were then performed using GoTaq DNA Polymerase (Promega #M7123) and the following primers (Sigma): *i)* for the Celf1 mutant allele (expected size 690 bp), Celf1-ko-mut-F 5′- GAA TTA TGG CCC ACA CCA GT-3′ and Celf1-ko-mut-R 5′-GAG GGT TTT GGC TCC TAT CC-3′ and *ii)* for the wild type Celf1 allele (expected size 490 bp), Celf1-ko-wt-F 5′-GGA CCA CCA GAG CTA CAG ACA-3′ and Celf1-ko-wt-R 5′-ACC ACC CAG ACC AAC CAG AT-3′. In both cases we multiplexed these PCRs using beta casein as an internal loading control with the following primers: Csn2-F 5′-GAT GTG CTC CAG GCT AAA GTT-3′ and Csn2-R 5′-AGA AAC GGA ATG TTG TGG AGT-3′ (expected band size 525 bp). For gender determination of postnatal mice we used the following set of primers in combination with the Csn2-F and Csn2-R for internal loading control: SRYdn 5′-GAG TAC AGG TGT GCA GCT CTA-3′ and SRYup 5′- CAG CCC TAC AGC CAC ATG AT-3′ (male expected size band: 450 bp). PCR program contained the following steps: *(i)* 95 °C for 1 min, *(ii)* 30 cycles of 95 °C for 30 s, 60 °C for 30 s and 72 °C for 30 s, *(iii)* 72 °C for 5 min, and *(iv)* 25 °C for 10 min. Amplification products were analyzed by 1% (mutant band) or 2% (wild type band and gender determination) agarose gel electrophoresis.

### RNA-seq experiments

RNA-seq samples were analyzed for quality and only those samples passing the following criteria were used for RNA-seq: *i)* RNA integrated number (RIN) ≥ 8.6, *ii)* ratio A_260nm_/A_280nm_ ≥ 1.8, *iii)* ratio A_260nm_/A_230nm_ ≥ 1.4, and *iv)* ratio r28S/16S ≥ 1.5. RNA sample preparation for sequencing was performed using Illumina TruSeq protocols as previously described^16^. A paired-end 100 cycles run was used to sequence the flow-cell on a HiSeq2000 Sequencing System

### Computational processing of RNA-seq data

Paired-end reads were aligned to the mouse genome (mm9) using TopHat 2.0.5^38^. RSEM^39^ was used for differentially expressed gene analysis and FPKM calculation. In this manner we computed the number of fragments that mapped into Ensembl gene models, and followed by edgeR^40^ we obtained the differentially expressed genes from those showing a false discovery rate FDR < 0.05. Gene expression was quantified by FPKM^41^. Differential alternative splicing events were identified utilizing the SpliceTrap^42^ that is based on Ensemble 65 gene model. Alternative splicing was quantified by the percentage of mRNAs containing an alternative region known as psi value^37^. The events with psi changes between two conditions |Δpsi| ≥ 15% were considered differential splicing events.

### Gene ontology analysis

The Database for Annotation, Visualization and Integrated Discovery (DAVID) v6.7^43^,^44^ was used for gene ontology analysis. We considered significant *p* values ≤ 0.05.

### Motif analysis

MEME version 4.9.0^26^ (motif>6–15 bp) software was used for motif analysis (maximum number of motifs was set at ten, and any number of repetitions was allowed). The E-value is the enrichment of a motif based on the following parameters: background frequencies, log likelihood ratio, width, sites, and training set size.

### Celf1 HITS-Clip data analysis

Celf1 HITS-CLIP data are available from the murine C2C12 cell line^28^. These Celf1 HITS-CLIP data were downloaded from the European Nucleotide Archive (ENA, accession number ERP00078912). The 4-bp tags were trimmed and we removed the sequences composed primarily of Illumina adapter. These pre-processed reads were then aligned to the mouse genome (mm9) using Bowtie13 software^45^ (allowing two mismatches). The reads with identical 5′ starts were collapsed into a single read to avoid potential PCR duplicates. Therefore, only unique mapped reads were considered Celf1 binding CLIP tags.

### Electrocardiograms and echocardiograms

ECGs and echocardiograms were performed in the Mouse Phenotyping Core at Baylor College of Medicine. Echocardiograms were performed using a Vevo 770 Visualsonics high-resolution ultrasound system with a 707B probe for the cardiac analysis. The animals were anesthetized with 2.5% isoflurane mixed with 100% oxygen and maintained with 1.0–1.5% isoflurane mixed with 100% oxygen during imaging. Images were obtained by an experienced imager in the short axis confirmation. All data was quantified using the Visualsonics Vevo analysis software package. Electrocardiograms were measured using the ECGenie (MouseSpecifics) equipment. Mice were placed atop the ECGenie pad and were allowed to acclimate for 5–7 min before data collection. Two segments of ECG data were then obtained for each mouse. All data was analyzed offline using the ECGenie software package.

### Statistics

Results are expressed as the mean ± s.e.m, Except for the *χ*^2^ test shown in Supplementary Table S1, *p*-values were estimated by the Student´s T-test (two tails). *p* ≤ 0.05 was considered significant.

## Additional Information

**Accession codes:** RNA-seq accession data: GSE85646.

**How to cite this article**: Giudice, J. *et al*. Neonatal cardiac dysfunction and transcriptome changes caused by the absence of Celf1. *Sci. Rep.* **6**, 35550; doi: 10.1038/srep35550 (2016).

## Supplementary Material

Supplementary Information

Supplementary Table S7

Supplementary Table S9
