# Supplementary material for: Neonatal cardiac dysfunction and transcriptome changes caused by the absence of Celf1
Source: Sci Rep. 2016 Oct 19;6:35550. doi: 10.1038/srep35550 (PMC5069560; doi:10.1038/srep35550)

Neonatal cardiac dysfunction and transcriptome changes caused by the absence of Celf1

Jimena Giudice[1,2], Zheng Xia[3,4], Wei Li[3,4], Thomas A. Cooper[1,3,5*]

1. Department of Pathology and Immunology, Baylor College of Medicine, Houston, TX, 77030, USA. **2.** Department of Cell Biology and Physiology, School of Medicine, University of North Carolina at Chapel Hill, NC, 27599, USA. **3.** Department of Molecular and Cellular Biology, Baylor College of Medicine, Houston, Texas, 77030, USA. **4.** Division of Biostatistics, Dan L Duncan Cancer Center, Baylor College of Medicine, Houston, TX, 77030, USA. **5.** Department of Molecular Physiology and Biophysics, Baylor College of Medicine, Houston, TX, 77030, USA.

* correspondence tcooper@bcm.edu

Supplementary information

Supplementary figure legends

Supplementary Figure S1. Celf1 protein levels. Celf1 protein levels were evaluated by Western blot assays at PN3 and PN38-42. Longer exposure of blot shown in **Fig. 1a** was used for detection of adult protein levels. All lanes are from the same blot. Brightness and contrast adjustments were equally applied to the whole blot. Cropped blots are displayed. Full-length blots are shown in **Supplementary Fig. S6**. Celf1 levels were estimated by densitometry and normalized to sarcomeric alpha actinin. Results are shown as mean ± s.e.m. *n* = 2.

Supplementary Figure S2. M-mode images examples. M-mode images from each genotype are consistent with the quantitative data presented in **Fig. 2b** and **Supplementary Table S5** in that there is little difference between the genotypes in the gross anatomic changes during contraction.

Supplementary Figure S3. Validation of alternative splicing RNA-seq data. a-b. Celf1 responsive splicing events identified by RNA-seq were validated by RT-PCR

assays. Left panel (**a**) shows events where Celf1 deletion induced more inclusion and right panel (**b**) shows events where Celf1 deletion promoted skipping (see **Supplementary Table S8**). Results are shown as mean ± s.e.m. * $p \leq 0.05$ Student t-test (2 tails), $n = 3$.

Supplementary Figure S4. GU rich motifs and Celf1-CLIP tags around alternative splicing events. a. Motif analysis was performed for 22 alternative splicing events regulated by Celf1 depletion using MEME software version 4.9.0. **b.** We analyzed the presence of the GU rich motif (#1) within the alternative spliced regions +/- 500 bp flanking sequences. **c.** We analyzed the presence of Celf1-CLIP tags (Masuda et al., 2012) around the 45 alternative splicing events regulated by Celf1 depletion in neonates (PN3): *i)* within the alternative region +/- 500 bp (blue), or *ii)* within a region between 500-800 bp (light blue).

Supplementary Figure S5. Genotypes of animals not selected for euthanasia suggest reduced survival of *Celf1*-/- animals.

Supplementary Figure S6. Full-length Western blots from Fig. 1. Images from full-length blots shown in **Fig. 1**.

Supplementary Figure S7. Full-length Western blots from Supplementary Fig. S1. Images from full-length blots shown in **Supplementary Fig. S1**. In red we labeled the lanes cropped for **Supplementary Fig. S1**.

Supplementary Figure S8. Full-length gels from Fig. S4. Images from full-length gels shown in **Fig. 4**.

Supplementary tables

Supplementary Table S1

Age	Genotype	Observed (O)	Expected (E)	$\frac{(O - E)^2}{E}$	χ^2
**PN3**	**-/-**	**12**	**18.75**	**2.4**	**3.2**
**PN3**	**+/-**	**42**	**37.50**	**0.5**	
**PN3**	**+/+**	**21**	**18.75**	**0.3**	
**PN3**	**Total**	**75**			
**PN21-28**	**-/-**	**10**	**26.75**	**10.5**	**14.0 ***
**PN21-28**	**+/-**	**65**	**53.40**	**2.5**	
**PN21-28**	**+/+**	**32**	**26.75**	**1.0**	
**PN21-28**	**Total**	**107**			

Supplementary Table S1. Genotype analysis by χ^2 test. The χ^2 test was performed on ten neonatal litters at PN3 and 15 litters at PN21-28. *$p < 0.001$ (degrees of freedom = 2).

Supplementary Table S2

genotype		HR bpm	PR ms	QRS ms	QT ms
**-/-**	**mean**	**469**	**0.106**	**0.056**	**0.158**
**+/-**	**mean**	**452**	**0.107**	**0.058**	**0.160**
**+/+**	**mean**	**427**	**0.101**	**0.059**	**0.163**
**-/-**	**s.d**	25	0.005	0.004	0.008
**+/-**	**s.d**	25	0.008	0.004	0.007
**+/+**	**s.d**	33	0.003	0.003	0.013
**-/-**	**s.e.m**	11	0.002	0.002	0.004
**+/-**	**s.e.m**	11	0.004	0.002	0.003
**+/+**	**s.em**	16	0.001	0.001	0.006
**-/-**	***n***	5	5	5	5
**+/-**	***n***	5	5	5	5
**+/+**	***n***	4	4	4	4
p -/- vs +/+		0.14	0.26	0.57	0.08
p -/- vs +/-		0.21	0.76	0.74	0.27
p +/+ vs +/-		0.83	0.49	0.69	0.32

Supplementary Table S2. ECG measurements at PN35-36. Animals were evaluated by ECG studies at 35-36 days of age and measurements were repeated 13 days later. Results were averaged. HR: heart rate.

Supplementary Table S3

genotype		D;s mm	D;d mm	SV vol	EF %	FS %	IVSd mm	LVIDd mm	LVPWd mm	IVSs mm	LVIDs mm	LVPWs mm
**-/-**	**mean**	**2.5**	**3.5**	**28**	**55**	**28**	**0.55**	**3.4**	**0.70**	**0.64**	**2.5**	**0.92**
**+/-**	**mean**	**2.6**	**3.7**	**33**	**56**	**29**	**0.55**	**3.7**	**0.79**	**0.94**	**2.6**	**1.04**
**+/+**	**mean**	**2.6**	**3.8**	**36**	**59**	**31**	**0.58**	**3.8**	**0.73**	**0.68**	**2.6**	**1.00**
**-/-**	**s.d**	0.1	0.1	2	3	2	0.1	0.1	0.1	0.1	0.1	0.1
**+/-**	**s.d**	0.2	0.3	9	4	3	0.1	0.3	0.1	0.6	0.2	0.1
**+/+**	**s.d**	0.3	0.3	5	7	4	0.1	0.3	0.1	0.1	0.4	0.2
**-/-**	**s.e.m**	0.1	0.1	1	2	1	0.04	0.1	0.04	0.03	0.1	0.03
**+/-**	**s.e.m**	0.1	0.1	4	2	1	0.03	0.1	0.05	0.27	0.1	0.07
**+/+**	**s.em**	0.1	0.1	2	3	2	0.03	0.1	0.04	0.03	0.2	0.08
**-/-**	***n***	4	4	4	4	4	4	4	4	4	4	4
**+/-**	***n***	5	5	5	5	5	5	5	5	5	5	5
**+/+**	***n***	5	5	5	5	5	5	5	5	5	5	5
***p* -/- *vs* +/+**		0.52	0.07	0.01*	0.31	0.26	0.60	0.06	0.56	0.29	0.48	0.40
***p* -/- *vs* +/-**		0.27	0.24	0.30	0.77	0.69	0.92	0.18	0.17	0.32	0.28	0.16
***p* +/+ *vs* +/-**		0.97	0.64	0.51	0.40	0.38	0.58	0.63	0.34	0.39	0.97	0.68

Supplementary Table S3. Echocardiogram measurements at PN35-36. Animals were evaluated by echocardiograms at 35-36 days of age and measurements were repeated 13 days later. Results were averaged. * $p \leq 0.05$ Student t-test (2 tails). EF: ejection fraction. FS: fractional shortening. IVSd: interventricular septal thickness at diastole. IVSs: interventricular septal thickness at systole. LVIDd: left ventricle internal diameter at diastole. LVIDs: left ventricle internal diameter at systole. LVPWs: Left ventricular posterior wall end systole. LVPWd: left ventricular posterior wall end diastole.

Supplementary Table S4

genotype		HR bpm	HRV bpm	CV %	RR ms	PQ ms	PR ms	QRS ms	QT ms	ST ms	QTC ms	QT disp. ms
**-/-**	**mean**	**421**	**195**	**37**	**187**	**38**	**51**	**19**	**86**	**67**	**69**	**91**
**+/-**	**mean**	**417**	**50**	**12**	**150**	**35**	**47**	**16**	**74**	**59**	**61**	**53**
**+/+**	**mean**	**415**	**15**	**3**	**151**	**36**	**48**	**16**	**75**	**59**	**61**	**41**
**-/-**	**s.d**	145	212	40	34	15	15	3	12	10	3	25
**+/-**	**s.d**	34	92	21	16	6	5	1	9	8	5	14
**+/+**	**s.d**	86	28	5	33	5	8	4	18	15	8	10
**-/-**	**s.e.m**	72	106	20	17	7	8	2	6	5	1	12
**+/-**	**s.e.m**	10	28	6	5	2	2	0	3	2	1	4
**+/+**	**s.em**	33	10	2	13	2	3	2	7	6	3	4
**-/-**	***n***	4	4	4	4	4	4	4	4	4	4	4
**+/-**	***n***	11	11	11	11	11	11	11	11	11	11	11
**+/+**	***n***	7	7	7	7	7	7	7	7	7	7	7
***p* -/- *vs* +/+**		0.94	0.19	0.19	0.14	0.84	0.74	0.23	0.27	0.32	**0.04***	**0.02***
***p* -/- *vs* +/-**		0.96	0.27	0.29	0.12	0.76	0.58	0.11	0.15	0.21	**0.003***	**0.05***
***p* +/+ *vs* +/-**		0.95	0.25	0.23	0.95	0.74	0.58	0.58	0.91	0.99	0.94	**0.04***

Supplementary Table S4. ECG measurements in neonates. Animals were evaluated by ECG studies at PN3-5. HR: heart rate. HRV: heart rate variability. disp: dispersion. * $p \leq 0.05$ Student t-test (2 tails).

Supplementary Table S5

genotype		D;s mm	D;d mm	SV vol	EF %	FS %	IVSd mm	LVIDd mm	LVPWd mm	IVSs mm	LVIDs mm	LVPWs mm
**-/-**	**mean**	**1.2**	**1.7**	**5.0**	**63**	**31**	**0.31**	**1.65**	**0.44**	**0.38**	**1.2**	**0.59**
**+/-**	**mean**	**1.1**	**1.7**	**5.8**	**69**	**36**	**0.36**	**1.67**	**0.44**	**0.46**	**1.2**	**0.64**
**+/+**	**mean**	**1.0**	**1.7**	**5.9**	**75**	**41**	**0.31**	**1.64**	**0.48**	**0.37**	**1.0**	**0.69**
**-/-**	**s.d**	0.1	0.1	0.3	7	4	0.05	0.08	0.08	0.05	0.1	0.06
**+/-**	**s.d**	0.2	0.3	1.8	8	6	0.06	0.25	0.06	0.13	0.5	0.09
**+/+**	**s.d**	0.2	0.2	1.8	7	6	0.03	0.23	0.09	0.02	0.2	0.13
**-/-**	**s.e.m**	0.1	0.1	0.2	3	2	0.02	0.04	0.04	0.03	0.1	0.03
**+/-**	**s.e.m**	0.1	0.1	0.5	2	2	0.02	0.08	0.02	0.04	0.1	0.03
**+/+**	**s.em**	0.1	0.1	0.7	3	2	0.01	0.09	0.03	0.01	0.1	0.05
**-/-**	***n***	4	4	4	4	4	4	4	4	4	4	4
**+/-**	***n***	11	11	11	11	11	11	11	11	11	11	11
**+/+**	***n***	7	7	7	7	7	7	7	7	7	7	7
***p* -/- *vs* +/+**		0.17	0.89	0.22	0.03*	0.02*	0.87	0.92	0.43	0.78	0.14	0.11
***p* -/- *vs* +/-**		0.52	0.58	0.18	0.18	0.12	0.10	0.82	0.95	0.12	0.68	0.22
***p* +/+ *vs* +/-**		0.38	0.58	0.87	0.14	0.14	0.03	0.81	0.33	0.05	0.19	0.43

Supplementary Table S5. Echocardiogram measurements in neonates. Animals were evaluated by echocardiograms at PN3-5. * $p \leq 0.05$ Student t-test (2 tails). EF: ejection fraction. FS: fractional shortening. IVSd: interventricular septal thickness at diastole. IVSs: interventricular septal thickness at systole. LVIDd: left ventricle internal diameter at diastole. LVIDs: left ventricle internal diameter at systole. LVPWs: Left ventricular posterior wall end systole. LVPWd: left ventricular posterior wall end diastole.

Supplementary Table S6

Geno-type	Age	Gender	Repli-cate	Read length	Total reads (pairs)	Mapped reads	Mapping rate
***Celf1* +/+**	PN38	Male	1	101 x 2	205,649,246	177,147,986	86%
***Celf1* +/+**	PN3	Male	1	101 x 2	190,063,901	162,369,900	85%
***Celf1* +/+**	PN3	Male	2	101 x 2	179,302,453	152,808,427	85%
***Celf1* -/-**	PN3	Male	1	101 x 2	192,429,400	165,348,253	86%
***Celf1* -/-**	PN3	Male	2	101 x 2	202,874,260	173,407,349	85%

Supplementary Table S6. RNA-seq summary. Overall parameters of the RNA-seq experiments

Supplementary Table S7 (excel). Alternative splicing RNA-seq data. Alternative splicing events regulated at PN3 in response to Celf1 depletion (*Celf1 -/-* versus *Celf1 +/+*). Alternative splicing events regulated during development in *Celf1 +/+* animals (PN38 *versus* PN3).

Supplementary Table S8

Gene symbol / alternative splicing event	Size region (nt)	Primer sequence (5' -3')		Band sizes (bp)	Δpsi KO - WT	
		Forward	Reverse		RNA-seq	RT-PCR
**Anks1 /** chr17:28195234-28199545	99	CTCTGCAGCTT CGATGATTG	GCCTCTTGGT CAGAATCAGG	204 / 105	25.4	9.5
**Aplp2 /** chr9:30972127-30976501	168	AGGGCACCATT TCAGACAAG	TCATTATCATC CGCTGAGGTC	277 / 109	-32.6	-10.9
**Atg13 /** chr2:91522182-91524916	99	CCCGTCAGTGG AAGACTCTC	AGGACAGCAG GCTGATAGGA	201 / 102	-28.6	-7.7
**Cd99l2 /** chrX:68694248-68703848	69	CTCGTTTGCCT CGTTTTCTC	TCCAAGGCAT CTTCCAAGTC	281 / 212	24.0	20.4
**Ivd /** chr2:118702623-118703683	105	CCACACCATTC CCTACTTGC	TAGACACTGAA TGCCGTCCA	239 / 134	-46.1	-38.7
**Ktn1 /** chr14:48323861-48325475	69	GATGGACAGAT CAAGTCTGTGG	GTTCTGCAGC TCCTGGACTT	183 / 114	53.5	29.8
**Lims1 /** chr10:57881146-57887441	188	TGGTCTCTGCT CTCAACAAGG	TGGCTTCATGT CAAATTCCA	295 / 107	21.8	5.2
**Macf1 /** chr4:123117792-123122195	62	CAAGGGCACTG CCTTAGAAA	TGAAGGCTTTC CTGTCGTTC	219 / 156	46.0	48.5
**Mapk8ip3 /** chr17:25055032-25060852	24	GAAAGATCCAA GATGCAGCAA	CAGGGGGAAG ACATTCAGAG	129 / 105	30.0	32.8
**Mical3 /** chr6:120902392-120907811	51	GGACTCAGACC TGTCCAGTGA	TTCCTCCTGCT TAGCCTGTC	199 / 148	38.2	24.5
**Myom1 /** chr17:71430113-71433774	294	TGAATGCAGCT GGACTTAGC	CCATCCAAGA ACCATGGAGT	428 / 134	51.3	8.6
**Nfya /** chr17:48534974-48539905	84	GGCCATGGAGC AGTATACGA	TAAGCTGGCC TCCACTGACT	209 / 125	-28.0	-29.6
**Rmnd1 /** chr10:5917369-5919018	97	ATTTCACAGCC CTGACTTCC	GCCTTTGCTG ATATGCTGTG	215 / 175 / 118	-21.1	-16.7
**Tmed2 /** chr5:124992980-124997012	21	GGATGTCCACT ATGACTCCAA	TCCCGGACTT CCATGTACTC	196 / 175	-0.5	-1.0
**Tpd52 /** chr3:8934991-	64	CCAAAAAGCTG GAAGACGTG	GGCTTGGCTC CTCCTACTTT	163 / 136 / 121 / 94	-30.8	-10.8

8944712						
**Ubn1 /** chr16:5081510-5086382	90	AGGATGCCATT GTCACAGGT	TTCCATCCTCC AAAGTGGTC	215 / 125	39.7	31.4

Supplementary Table S8. AS events validated. Alternative splicing events were validated by RT-PCR using heart samples from *Celf1 +/+* (WT) and *Celf1 -/-* (KO) animals at PN3 and adult stages.

Supplementary Table S9 (excel). Gene expression RNA-seq data. Gene expression data for PN3 hearts in the presence or absence of Celf1 (*Celf1 -/-* versus *Celf1 +/+*). Gene expression changes during development in *Celf1 +/+* animals (PN38 versus PN3).

Supplementary Table S10

Gene ontology term	#	p	Genes
**cell division**	24	4E-15	*Cdc7, Cdk1, Prc1, Ccnf, Nusap1, Cenpe, Ndc80, Aurkb, Cep55, Ube2c, Smc2, Fam83d, Ccnb1, Ccne2, Spc25, Spag5, Oip5, Incenp, Fbxo5, Ccna2, Top2a, Cdca5, Hells, Ercc6l*
**mitotic cell cycle**	22	3E-14	*Cdk1, Ccnf, Skp2, Nusap1, Cenpe, Ndc80, Aurkb, Cep55, Ube2c, Smc2, Fam83d, Ccnb1, Spc25, Spag5, Oip5, Incenp, Bcl2, Fbxo5, Ccna2, Cdca5, Hells, Ercc6l*
**cell cycle**	31	2E-13	*E2f2, Prc1, E2f7, Aurkb, Cep55, Fam83d, Ccne2, Spc25, Oip5, Bcl2, Incenp, Fbxo5, Ccna2, Cdca5, Hells, Ercc6l, Exo1, Cdc7, Cdk1, Ccnf, Skp2, Nusap1, Ndc80, Cenpe, Tacc3, Ube2c, Smc2, Ccnb1, Sass6, Spag5, Chaf1b*
**M phase**	22	5E-13	*Exo1, Cdk1, Ccnf, Nusap1, Cenpe, Ndc80, Aurkb, Cep55, Tacc3, Ube2c, Smc2, Fam83d, Ccnb1, Spc25, Spag5, Oip5, Incenp, Fbxo5, Ccna2, Cdca5, Hells, Ercc6l*
**microtubule-based process**	15	2E-08	*Kif23, Tubb2a, Kif5c, Kif15, Kif18b, Nusap1, Cenpe, Ndc80, Tacc3, Spc25, Kif1a, Kif4, Fbxo5, Tuba1b, Tuba1c*
**chromosome segregation**	7	4E-05	*Spc25, Incenp, Nusap1, Ndc80, Cenpe, Cdca5, Top2a*
**cell cycle reg.**	11	5E-05	*Ccne2, Cdk1, E2f2, Plk4, Bcl2, Btc, Skp2, Fbxo5, Nusap1, Cenpe, Tacc3*
**microtubule cytoskeleton org.**	7	8E-04	*Spc25, Fbxo5, Nusap1, Ndc80, Cenpe, Tacc3, Tuba1b*
**chromosome condensation**	4	1E-03	*Nusap1, Smc2, Cdca5, Top2a*
**cytoskeleton org.**	11	1E-03	*Spc25, Bcl2, Capza1, Fbxo5, Ush1c, Nusap1, Ndc80, Cenpe, Cnn1, Tacc3, Tuba1b*
**cytokinesis**	4	2E-03	*Prc1, Incenp, Nusap1, Aurkb*
**spindle org.**	4	3E-03	*Spc25, Fbxo5, Ndc80, Tacc3*
**DNA packaging**	6	3E-03	*Hist1h2bk, Nusap1, Smc2, Cdca5, Top2a, Hells*
**steroid biosynthesis**	5	5E-03	*Hsd17b12, Idi1, Hsd17b7, Sc4mol, Dhcr24*
**protein polymerization**	4	7E-03	*Tubb2a, Fbxo5, Tuba1b, Tuba1c*
**apoptosis reg.**	8	9E-03	*Stil, Bcl2, Btc, Skp2, Bnip3, Hells, Angptl4, Dhcr24*
**monosaccharide catab**	4	2E-02	*Ldha, Gnpda1, Pgam1, Rpia*
**stress response**	10	2E-02	*Exo1, Cdk1, Neil3, Zmat3, Bcl2, Eme1, Chaf1b, Gm14378, Mif, Angptl4*
**cholesterol biosynthesis**	3	2E-02	*Idi1, Hsd17b7, Dhcr24*
**carbohydrate catab.**	4	2E-02	*Ldha, Gnpda1, Pgam1, Rpia*
**protein amino acid phosphorylation**	13	2E-02	*Cdc7, Bmp10, Cdk1, Camk1g, Ttk, Aurkb, Pbk, Ccnb1, Hunk, Plk4, Bcl2, Ripk3, Gm14378*
**hexose metab.**	6	3E-02	*Ldha, Gnpda1, Pgam1, Gale, Rpia, B3galtl*
**cofactor metab.**	6	3E-02	*Mthfd2, Gsr, Itgb1bp3, Spna1, Rpia, Mthfd1l*
**cell proliferation reg.**	11	4E-02	*Bmp10, Odc1, Bcl2, E2f7, Edn1, Serpine1, Btc, Bex1, Bmp7, Etv4, Mif*
**growth**	6	4E-02	*Stil, Bmp10, Inhba, Bcl2, Bmp7, Gap43*
**coenzyme metab.**	5	5E-02	*Mthfd2, Gsr, Itgb1bp3, Rpia, Mthfd1l*

Supplementary Table S10. Gene ontology analysis of the up-regulated genes in *Celf1 -/-* animals. Summary of the gene ontology analysis (biological processes) performed using DAVID software for the genes up-regulated (≥ 1.5 fold change, FDR ≤ 0.05) in PN3 *Celf1-/-* hearts. catab: catabolism. metab: metabolism. org: organization. reg: regulation.

Supplementary Table S11

Gene ontology term	#	*p*	Genes
**response to virus**	6	2E-03	*Ifih1, Kcnj8, Ifi27l2a, Irf7, Ticam1, Rsad2*
**metal ion transport**	14	3E-03	*Steap4, Hcn2, Kcnd2, Kcnb1, Cacng6, Kcnip2, Kcnj8, Atp2a3, Cacna1g, Cacna1h, Camk2b, Scara5, Slc40a1, Scn4a*
**cation transport**	15	4E-03	*Hcn2, Steap4, Atp5e, Kcnd2, Kcnb1, Cacng6, Kcnip2, Kcnj8, Atp2a3, Cacna1g, Cacna1h, Camk2b, Scara5, Slc40a1, Scn4a*
**immune effector process**	7	4E-03	*C1ra, Kcnj8, Irf7, Ticam1, Rsad2, C1s, Dbh*
**intracel. receptor-mediated signaling**	4	7E-03	*Rarg, Klf9, Rxrg, Calcoco1*
**circadian rhythm**	4	1E-02	*Nr1d1, Dbp, Per1, Per3*
**immune response**	13	1E-02	*Ifih1, Mill2, Gbp9, Rsad2, Vtn, C1s, Dbh, C1ra, Kcnj8, Irf7, Ticam1, Eda, Gbp3*
**heterocycle catabolism**	4	2E-02	*Dpyd, Dbh, Aldh1l2, Entpd2*
**rhythmic process**	5	3E-02	*Nr1d1, Dbp, Tef, Per1, Per3*
**oxidation reduction**	15	3E-02	*Bckdha, Phyhd1, Sqrdl, Steap4, D2hgdh, Me3, Maob, Aldh1l2, Dbh, Sod3, Msrb2, Ivd, Fmo1, Dpyd, Alox5*
**potassium ion transport**	6	4E-02	*Hcn2, Kcnd2, Kcnj8, Kcnb1, Kcnip2, Scn4a*
**transmembrane transport**	11	5E-02	*Slc2a12, Hcn2, Atp5e, Kcnd2, Abcb1a, Kcnb1, Cacna1h, Aqp7, Aqp1, Scara5, Scn4a*

Supplementary Table S11. Gene ontology analysis of the down-regulated genes in *Celf1 -/-* animals. Summary of the gene ontology analysis (biological processes) performed using DAVID software for the genes down-regulated (≥ 1.5 fold change, FDR ≤ 0.05) in PN3 *Celf1-/-* hearts.

Supplementary Table S12

Cell cycle genes regulated by *Celf1 -/-* at PN3 (group a)				Genes unaffected by *Celf1 -/-* at PN3 (group b)			
Gene symbol	Celf1-CLIP tags			Gene symbol	Celf1-CLIP tags		
	3'UTR	intron / exon	total		3'UTR	intron / exon	total
E2f2	11	3	14	***Muted***	0	8	8
Prc1	21	7	28	***Rer1***	2	5	7
E2f7	16	11	27	***Kdr***	1	8	9
Aurkb	16	3	19	***Osbp***	11	9	20
Cep55	3	11	14	***Atp6v0a1***	0	14	14
Fam83d	7	3	10	***Map2k2***	8	0	8
Ccne2	2	3	5	***Atox1***	0	0	0
Spc25	23	2	25	***Ifit2***	2	3	5
Oip5	6	13	19	***Dhrs1***	2	1	3
Bcl2	2	51	53	***Grcc10***	4	12	16
Incenp	16	17	33	***Bckdk***	0	0	0
Fbxo5	16	3	19	***Mrpl54***	0	0	0
Ccna2	17	0	17	***Adrbk1***	6	3	9
Cdca5	0	1	1	***Atg12***	8	0	8
Hells	19	34	53	***Psmb6***	0	0	0
Ercc6l	4	9	13	***Gm10094***	7	1	8
Exo1	0	9	9	***H47***	0	1	1
Cdc7	1	8	9	***Tmod1***	2	9	11
Cdk1	15	5	20	***Tspan9***	1	35	36
Ccnf	9	8	17	***Snx21***	0	0	0
Skp2	8	8	16	***Ei24***	26	11	37
Nusap1	23	15	38	***Mtmr6***	9	5	14
Ndc80	0	10	10	***Aars2***	0	4	4
Cenpe	0	9	9	***Cadm4***	0	13	13
Tacc3	3	4	7	***Rbms2***	4	17	21
Ube2c	25	2	27	***Ubl7***	0	9	9
Smc2	35	21	56	***Pisd***	1	9	10
Ccnb1	20	4	24	***Rit1***	5	4	9
Sass6	7	13	20	***Tmem182***	1	8	9
Spag5	0	1	1	***Chmp2b***	10	16	26
Chaf1b	0	5	5	***Dhrs11***	0	0	0
**Mean**	10	9	20	**Mean**	4	7	10
**s.e.m.**	9	10	14	**s.e.m**	5	7	10
***p* (group a *versus* group b)**	9E-04*	2E-01 (n.s)	3E-03*				

Supplementary Table S12. Analysis of Celf1-CLIP tags within cell cycle genes regulated by Celf1 depletion (group a) and control unaffected genes randomly selected (group b). Celf1-CLIP tags located within the 3'UTRs or intronic / exonic regions were computed for the 31 genes included in the "cell cycle" gene

ontology category and 31 unaffected control genes. Bottom part of the table shows results as mean and s.e.m. * $p \leq 0.05$ Student t-test (2 tails). n.s: not significant

Supplementary figures

Supplementary Figure S1

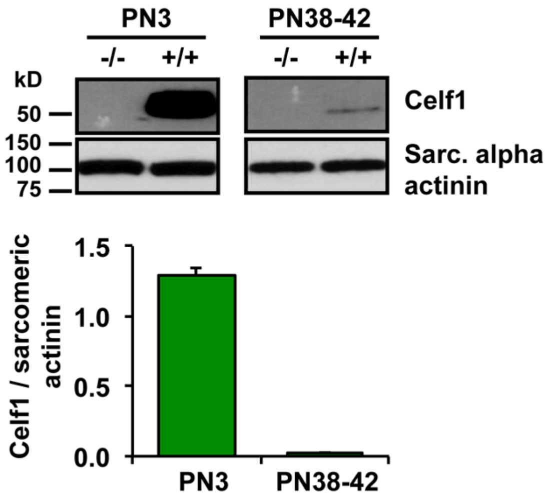

Supplementary Figure S2

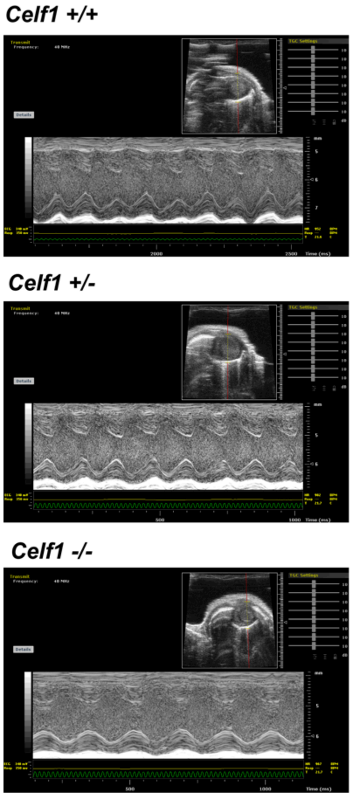

Supplementary Figure S3

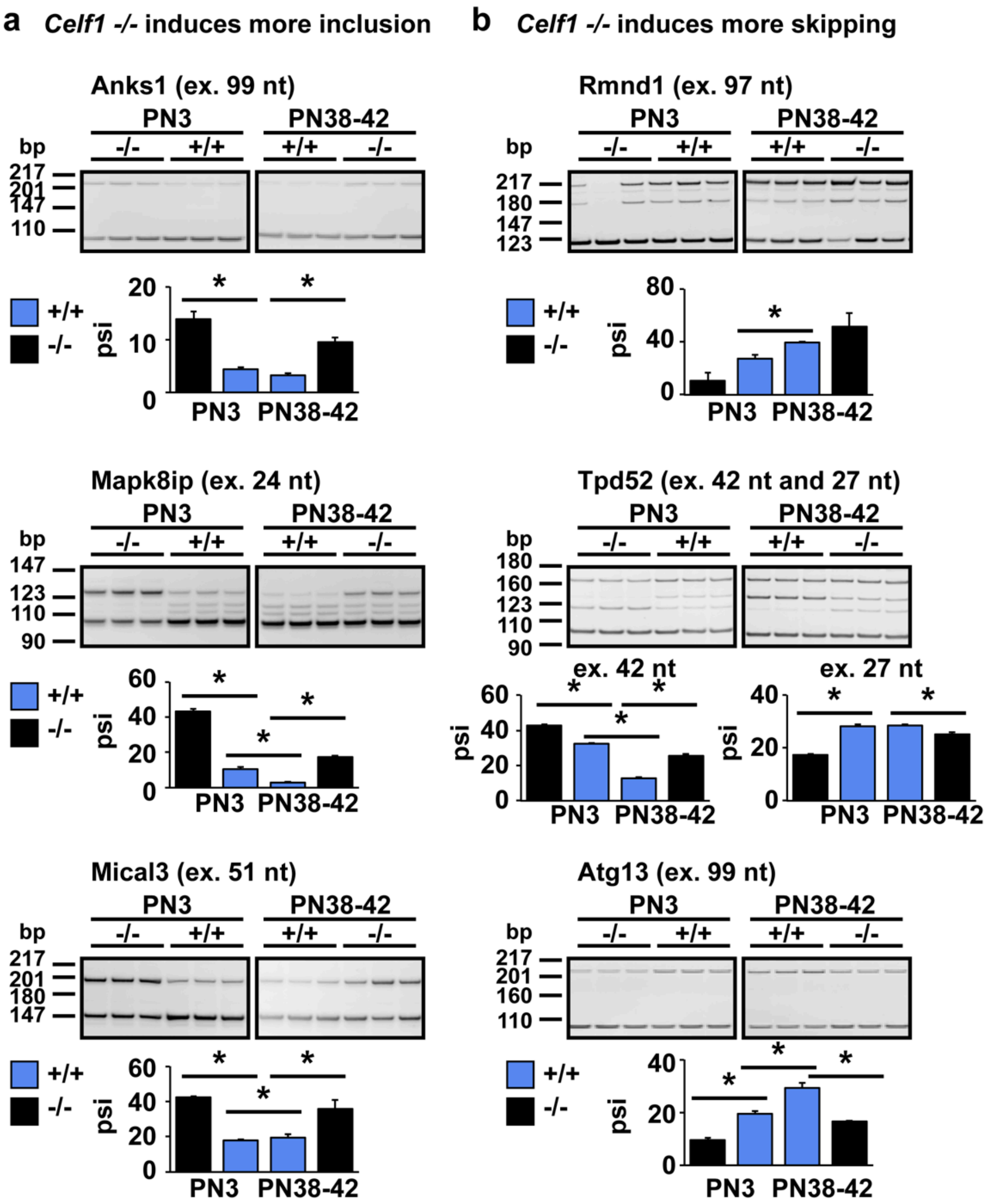

Supplementary Figure S4

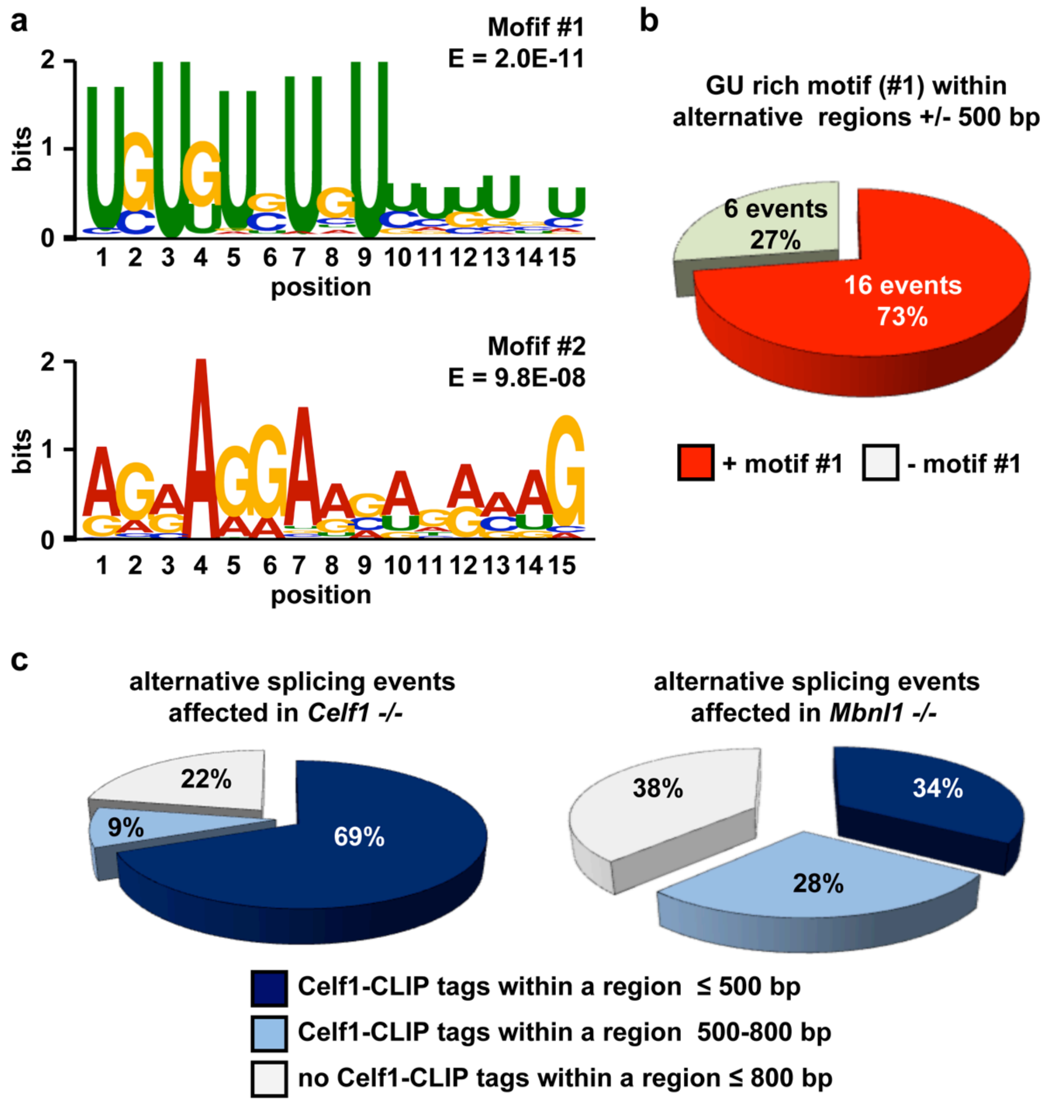

Supplementary Figure S5

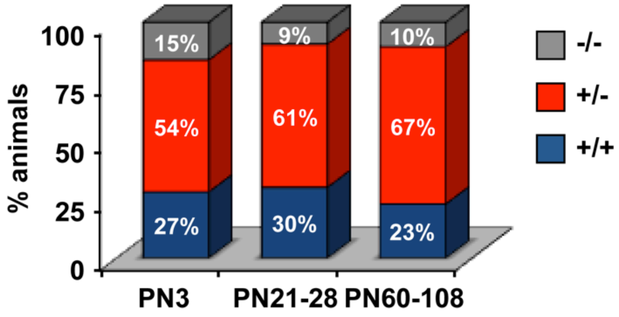

age genotyping	PN3	PN21-28	PN60-108
-/-	12 (15%)	10 (9%)	5 (10%)
+/-	42 (54%)	65 (61%)	32 (67%)
+/+	21 (27%)	32 (30%)	11 (23%)
total	78 (100%)	107 (100%)	48 (100%)

Supplementary Figure S6

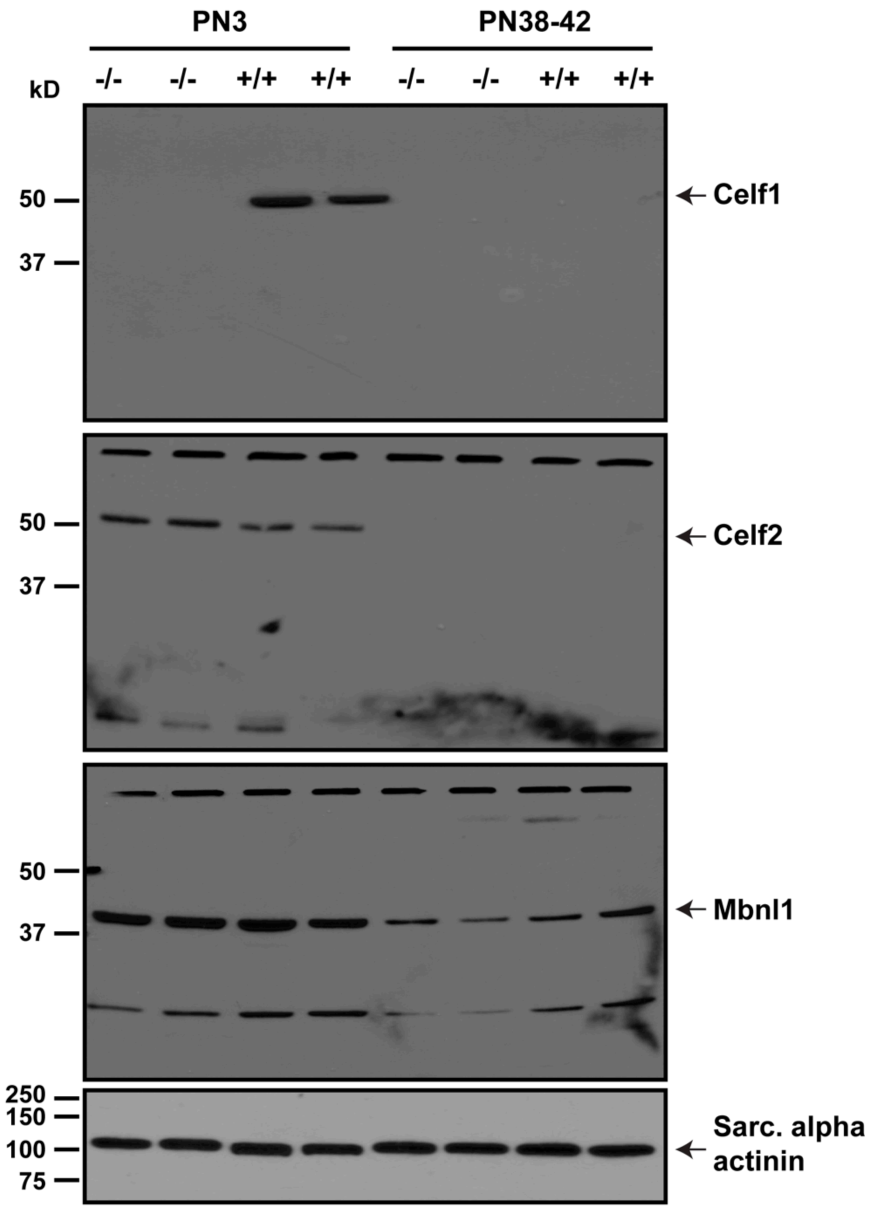

Supplementary Figure S7

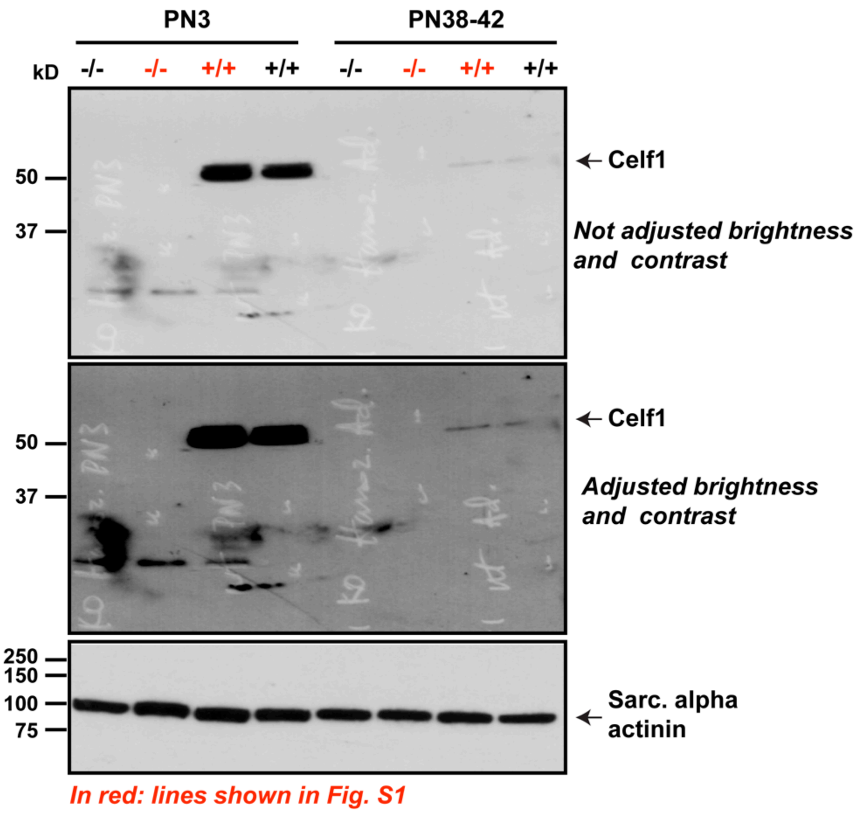

Supplementary Figure S8

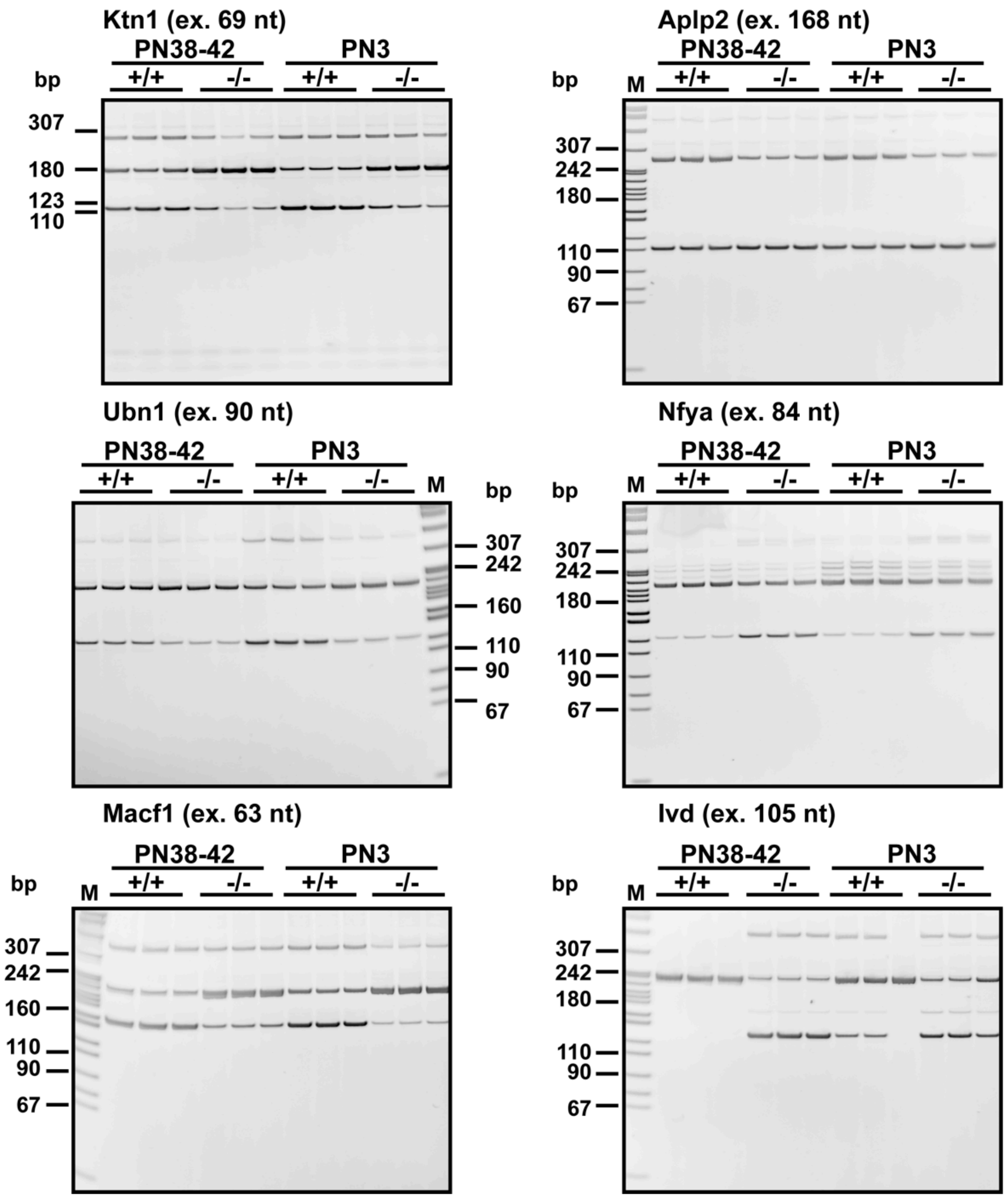

Supplement: Supplementary Information [file srep35550-s1.pdf]
